# Alternative Pharmacokinetic Metrics in Single-Dose Studies to Ensure Bioequivalence of Prolonged-Release Products at Steady State—A Case Study

**DOI:** 10.3390/pharmaceutics15020409

**Published:** 2023-01-26

**Authors:** Víctor Mangas-Sanjuán, Marta Simón, Esperanza González-Rojano, Dolores Ochoa, Francisco Abad-Santos, Manuel Román, Mercedes Ramos, Carlos Govantes, Alfredo García-Arieta

**Affiliations:** 1 Department of Pharmacy and Pharmaceutical Technology and Parasitology, University of Valencia, 46100 Valencia, Spain; 2 Interuniversity Research Institute for Molecular Recognition and Technological Development, Polytechnic University of Valencia—University of Valencia, 46100 Valencia, Spain; 3 Laboratorios Normon, 28760 Madrid, Spain; 4 Clinical Pharmacology Department, Hospital Universitario Clínico San Carlos, Instituto de Investigación Sanitaria del Hospital Clínico San Carlos (IdISSC), 28040 Madrid, Spain; 5 Clinical Pharmacology Department, Hospital Universitario de La Princesa, Instituto Teófilo Hernando, Instituto de Investigación Sanitaria la Princesa (IIS-IP), 28006 Madrid, Spain; 6 Pharmacology Department, Facultad de Medicina, Universidad Autónoma de Madrid, 28029 Madrid, Spain; 7 División de Farmacología y Evaluación Clínica, Departamento de Medicamentos de Uso Humano, Agencia Española de Medicamentos y Productos Sanitarios, 28022 Madrid, Spain

**Keywords:** bioequivalence, single dose, steady state, prolonged release, pAUC

## Abstract

(1) Background: this article investigates which PK metrics in a single-dose study (concentration at the end of posology interval, C_τ_, partial areas under the curve, pAUCs, or half-value duration, HVD) are more sensitive and less variable for predicting the failure of a prolonged-release product at steady-state that was the bioequivalent for C_max_, AUC_0-t_ and AUC_0-inf_, in the single-dose study; (2) Methods: a cross-over study was performed in 36 subjects receiving desvenlafaxine 100 mg prolonged-release tablets. Conventional (C_max_, AUC_0-t_ and AUC_0-inf_) and additional (C_τ_, pAUCs and HVD) PK metrics were considered after single-dose conditions. Predicted PK metrics at steady state (AUC_0-τ_, C_max,ss_, and C_τ,ss_) were derived using a population PK model approach; (3) Results: the existing differences in the shape of the concentration–time curves precluded to show equivalence for C_τ,ss_ in the simulated study at steady state. This failure to show equivalence at steady state was predicted by C_τ_, pAUCs and HVD in the single-dose study. C_τ_ was the most sensitive metric for detecting the different shape, with a lower intra-subject variability than HVD; (4) Conclusions: conventional PK metrics for single-dose studies (C_max_, AUC_0-t_ and AUC_0-inf_) are not enough to guarantee bioequivalence at steady state for prolonged-release products.

## 1. Introduction

For prolonged-release generic products, there is almost a worldwide consensus on the need for single-dose fasted and fed bioequivalence (BE) studies [1]. However, the need of BE studies at steady state for the approval of generic prolonged-release products that exhibit accumulation after repeated dosing is a controversial issue. While the EMA guideline on the pharmacokinetic and clinical evaluation of modified-release dosage forms [2] requires a study at steady state, the USFDA does not require such a study [3]. The USFDA justifies the absence of the requirement of a multiple-dose study at steady state: “Because single-dose studies are considered more sensitive in addressing the primary question of BE (e.g., release of the drug substance from the drug product into the systemic circulation), multiple-dose studies are generally not recommended” [3]. In the European Union, this is agreed for immediate release products [4,5,6,7,8,9,10,11,12,13,14], because the elimination of the drug is the same for test and reference, and is independent of the product. Consequently, the shape of the plasma concentration curve may differ during the absorption, which is addressed by C_max_ (and T_max_ or partial AUC (pAUC) when the onset of action is clinically relevant), but the shape of the final elimination phase has to be similar for test and reference. On the contrary, in the case of prolonged-release products, the absorption rate is slower than the elimination rate and, consequently, the shape of the final part of the curve depends on the absorption, which is product dependent. Therefore, the shape of the plasma concentration–time curves might differ notably and this difference would not be detected by C_max_, which is particularly insensitive due to the plateau shape of these profiles. Consequently, in the European Union, the conventional metrics C_max_, AUC_0-t_ and AUC_0-inf_ are not enough to characterize the shape of the plasma concentration–time curve in the case of prolonged-release products [2].

In the European Union, the requirements differ where the accumulation is considered significant or not [2]. Accumulation is considered significant where the mean AUC in the posology interval (AUC_0-τ_) after the first dose covers less than 90% of the mean AUC_0-inf_ for both test and reference. Where the accumulation is significant, a comparison at steady state is required. However, when the accumulation is not significant, the shape of the curves is compared with pAUCs as additional metrics. In this case, the pAUCs are not intended to assess the rate of absorption, but to assess the similarity in the shape of the curve, even when the release is not multiphasic. Therefore, the cut-off point is not defined at a sampling time with clinical relevance for the onset of action or at Tmax, but with the intention to split the AUC in two pieces (e.g., the cut-off point at half of the dosage interval is recommended, unless otherwise scientifically justified). However, in this case of insignificant accumulation, most of the exposure (AUC) will be observed in the first half of the posology interval and most samples are taken soon after administration. Therefore, the two pieces of AUC may be unbalanced, with lower levels and higher variability in the second half (e.g., melatonin prolonged-release products [15]. It might be convenient not to cut off the AUC based on the posology interval, but on the equal number of sampling times and the similarity of the extent of exposure, in order to reduce the variability.

In addition to pAUCs, some Product-Specific Bioequivalence Guidelines (e.g., octreotide) [16] also require the demonstration of bioequivalence in the single-dose study of some prolonged-release injectables with a significant accumulation for the concentration at the end of the posology or the dosing interval (C_τ_); this is in order to waive the multiple-dose study as it is extremely difficult to conduct, due to the long duration of the study, the insufficient recruitment of patients, and/or the unacceptable safety profile of the product at the steady state for healthy volunteers. Assuming linear pharmacokinetics (PK), the concentration at the end of the posology interval after a single dose has shown to be a potential surrogate to assess the shape of the plasma concentration–time curve, in order to avoid the need of a multiple-dose study [17]; however, it has been reported that C_τ_ is more variable than conventional metrics and is not always predictive of the C_τ_ at steady state (C_τ,ss_) [18]. Nonetheless, the multiple-dose study is still preferred in the EU [2]. A requirement based on C_τ_ could be extremely difficult to pass, as it seems to be highly variable and the multiple-dose study is more informative in case of any non-linearity [19].

The aim of the present study is to show the case of a prolonged-release generic product that was able to demonstrate bioequivalence in C_max_, AUC_0-t_ and AUC_0-inf_ in the single-dose fasted state study, as required by the USFDA [20], but whose development was abandoned because the differences in the shape of the curve anticipated a failure to show equivalence in the corresponding steady-state study, which is required in the European Union. Furthermore, this real case offers the possibility of investigating which shape parameters in the single-dose study (C_τ_, pAUCs or half-value duration (HVD)) are more sensitive and less variable for predicting the failure at steady state, as well as the most adequate cut-off value for the pAUCs.

## 2. Materials and Methods

### 2.1. Study Design

A phase I, single-dose, open-label, randomized cross-over bioequivalence study, with 2 treatments, 2 sequences and 2 periods, was performed in 36 healthy volunteers (18 men and 18 women) who received desvenlafaxine 100 mg prolonged-release tablets under fasting conditions in both periods (EudraCT number: 2019-000628-17). The inclusion criteria for the participants of the study were male or female subjects from 18 to 55 years of age, and free from organic or psychic conditions, with no clinically significant abnormalities in hematology, coagulation, biochemistry, serology and urinalysis. In total, 32 subjects (17 men and 15 women) competed the study and were included in the analysis.

The reference formulation (Pristiq^®^) was marketed by Laboratories Pfizer and the test formulation was manufactured by Laboratorios Normon (Spain). Each subject received one of these two desvenlafaxine 100 mg prolonged-release tablets by oral route in each period, as allocated by block randomization. Both treatment periods were separated by a 7-day washout period.

The final approved protocol and the Informed Consent Form were reviewed by the Independent Ethics Committee on Clinical Research (IECCR) of the “Hospital Universitario de La Princesa”. The date of approval was 28 May 2019. The study was also approved by the Spanish Agency for Medicines and Healthcare Products on 30 May 2019. The study was conducted in accordance with the SOPs of the Clinical Trials Unit of “Hospital Universitario de La Princesa”, in accordance with current Spanish regulations and the ICH guidelines for Good Clinical Practice (R2) [21], and performed according to the Revised Declaration of Helsinki [22].

### 2.2. Blood Sampling and Analytical Methods

For the characterization of the pharmacokinetic profile, blood samples were obtained pre-dose and at 1.0, 2.0, 3.0, 4.0, 5.0, 6.0, 6.5, 7.0, 7.5, 8.0, 8.5, 9.0, 10.0, 11.0, 12.0, 16.0, 20.0, 24.0, 48.0 and 72.0 hours after the administration of the products.

All blood samples were collected in 3 mL K_2_EDTA tubes by direct venipuncture or from an indwelling cannula, which were placed in an arm vein of the subject. The tubes were centrifuged at 4 °C for 10 min at 1900 G. Once centrifuged, 0.5 mL of plasma was aliquoted in these tubes and they were stored in a freezer at −2 °C ± 5 °C immediately.

The method involved a protein precipitation extraction procedure with 0.1% formic acid in methanol. Desvenlafaxine (O-Desmethylvenlafaxine) and internal standard were measured by reversed-phase high-performance liquid chromatography coupled to a tandem mass spectrometry detector (LC/MS/MS). The lower limit of quantification was 1.00 ng/mL. The accuracy and precision of the analytical method was 101.27% and 2.69%, respectively.

### 2.3. Pharmacokinetic and Statistical Analysis

The primary pharmacokinetic parameters: the area under the curve from time zero to the last measurable concentration (AUC_0-t)_, and from zero to infinity (AUC_0-inf_), as well as the maximum concentration (C_max_), were calculated for the 32 subjects that completed both periods, using non-compartmental analysis (NCA) with the linear trapezoidal rule. 

Standard BE assessment was conducted based on the 90% confidence interval (CI) of the geometric mean ratio (GMR) of these primary pharmacokinetic parameters. The BE between both products was declared according to the EMA requirements if the 90% CI for GMR of the test/reference products was contained within the acceptance range of 80.00–125.00%.

Alternatively, several PK metrics from the single-dose fasted-state study have been investigated as a surrogate for the performance in the multiple-dose study. In this sense, the following metrics have been calculated with NCA in order to evaluate their predictive performance in a multiple-dose study.

- C_τ_: the concentration at the end of the dosing interval in the single-dose study.
- pAUC_0–8h_ and pAUC_8h-t_: the partial areas under the curve with a cut-off point of 8 h, i.e., from 0 to 8 h (11 data points), and from 8 h to the last measurable concentration (11 data points).
- pAUC_0–10h_ and pAUC_10h-t_: the partial areas under the curve with a cut-off point of 10 h, i.e., from 0 to 10 h (14 data points), and from 10 h to the last measurable concentration (8 data points).
- pAUC_0–12h_ and pAUC1_12h-t_: the partial areas under the curve with a cut-off point of 12 h, i.e., from 0 to 12 h (16 data points), and from 12 h to the last measurable concentration (6 data points).
- pAUC_0–16h_ and pAUC1_16h-t_: the partial areas under the curve with a cut-off point of 16 h, i.e., from 0 to 12 h (17 data points), and from 12 h to the last measurable concentration (5 data points).
- pAUC_0–20h_ and pAUC1_20h-t_: the partial areas under the curve with a cut-off point of 20 h, i.e., from 0 to 12 h (18 data points), and from 12 h to the last measurable concentration (4 data points).
- pAUC_0–24h_ vs. pAUC_24h-t_: the partial areas under the curve with a cut-off point of 24 h, i.e., from 0 to 24 h (19 data points), and from 24 h to the last measurable concentration (3 data points).
- Half-value duration (HVD or T50%C_max_): the time span during which the plasma concentrations are equal to or higher than half of the C_max_ value [19], calculated according to the linear trapezoidal rule.

Pharmacokinetic parameters were calculated using Microsoft Excel 365^®^ and Phoenix 64 WinNonlin (8.3.4.295).

### 2.4. Prediction of the Multiple-Dose Fasted-State Study Using a Model-Based Approach

The time course of the 100 mg daily dose of desvenlafaxine in the multiple-dose fasted-state study at steady state were simulated using a population PK model, developed after single-dose conditions in these 32 healthy volunteers. The bioequivalence assessment was conducted using individual predicted concentrations at steady state. The modelling strategy first evaluated the PK models with different structural dispositions, including their linear distribution and elimination processes. Then, the model was adapted to describe the absorption kinetics of desvenlafaxine oral products by assuming linear absorption kinetics. Non-linear absorption kinetics, absorption latency and complex absorption processes (parallel absorption, precipitation, transit compartments, etc.) were also evaluated.

#### 2.4.1. Data Analysis

Observed plasma concentrations were logarithmically transformed. All data analyses were performed based on the population approach with the software NONMEM^®^ (v7.4, ICON plc Development Solutions, Hanover, MD, USA). The population PK parameters were estimated using the First-Order Conditional Estimation with Interaction method.

Inter-individual (IIV) and/or inter-occasion variability (IOV) associated with the PK model parameters were modeled exponentially, preventing negative values for the individual estimates. The residual unexplained variability (RUV) was described with an additive model on the logarithmic scale. The significance of the non-diagonal elements of the Ω variance–covariance matrix and the subject-specific RUV were also evaluated.

#### 2.4.2. Model Selection

Model selection was based on physiological and pharmacological rationale with the principle of parsimony [23]. The minimum value of the objective function value (OFV), provided by NONMEM^®^ and approximately equal to −2xlog (likelihood) (−2LL), and the Akaike Information Criteria (AIC), for nested and non-nested models, respectively, were used together with the visual inspection of the goodness of fit (GOF) plots to perform model selection. A decrease of 6.63 points in the -2LL value, between two nested models differing in one parameter, was considered significant at the 1% level.

#### 2.4.3. Model Evaluation

The model evaluation of the selected models was performed through prediction-corrected visual predictive checks (pc-VPC) [24]. Briefly, one thousand simulated datasets were simulated; the 2.5th, 50th, and 97.5th percentiles for every simulated study and sampling time-period were calculated. Then, the 95% prediction intervals of the percentiles were calculated and displayed graphically together, with the corresponding percentiles computed from raw data. In addition, the condition number, computed as the ratio of the largest eigenvalue to the smallest eigenvalue of the variance–covariance matrix, was calculated. A condition number lower than 400 was targeted, as it indicates good stability in the parameter estimates [25]. The model evaluation also included an assessment of the normalized prediction distribution errors (NPDE) reported from NONMEM [26]. The precision of the model parameter estimates, defined as the relative standard error (RSE), was calculated from the variance–covariance matrix (when possible) and from the analysis of one thousand simulated bootstrap datasets.

For graphical and statistical analysis, the R software (http://cran.r-project.org (accessed on 15 January 2021, version 4.0.3) was used. Pc-VPC and bootstrap analyses were performed using PsN [27].

## 3. Results

### 3.1. Single-Dose Fasted-State Study

**Figure 1:**
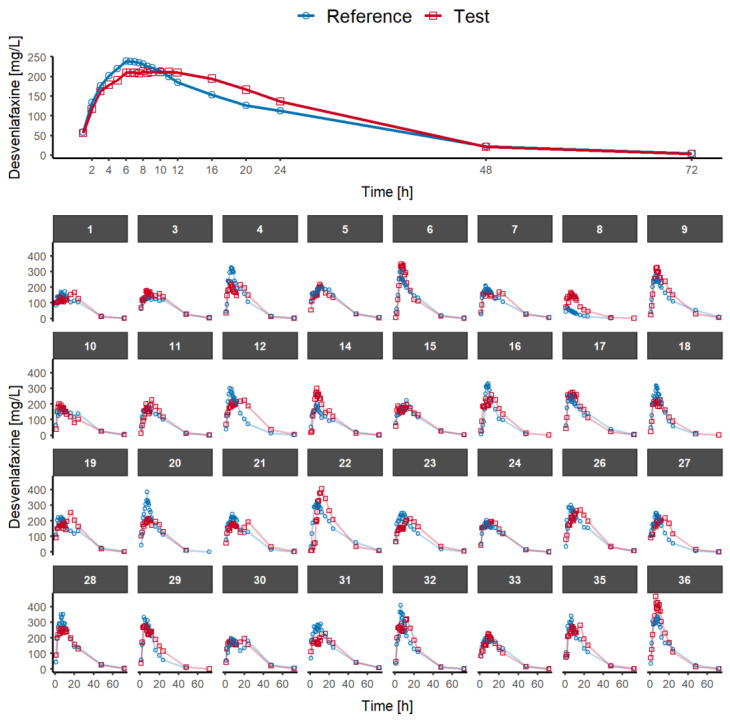
Average and individual observed plasma concentrations of desvenlafaxine vs. time after the administration of desvenlafaxine 100 mg prolonged-release tablets as a single dose in fasted conditions in 32 healthy volunteers. Blue circles and red squares represent the experimental desvenlafaxine observations of reference and the test formulations, respectively.

The average and individual observed plasma concentration–time profiles of desvenlafaxine in the 32 healthy volunteers that completed the study are depicted in Figure 1. The AUC_0-τ_, i.e., AUC_0–24h_, in the single-dose study represents less than 90% of AUC_0-inf_ (66% for test and 67% for the reference product). Therefore, the accumulation is considered to be significant.

Supplementary Table S1 includes the individual AUC_0-t_, AUC_0-inf_ and C_max_ values for each subject and treatment. The results of the BE assessment after a single dose in the fasted state are summarized in Table 1, showing that bioequivalence can be concluded. 

Supplementary Table S2a–e includes the individual values of C_τ_, pAUC_0–8h_, pAUC_8h-t_, pAUC_0–10h_, pAUC_10h-t_, pAUC_0–12h_, pAUC_12h-t_, pAUC_0–16h_, pAUC_16h-t_, pAUC_0–20h_, pAUC_20h-t_, pAUC_0–24h_, pAUC_24h-t_ and HVD for each subject and treatment. The results of the BE assessment after the single-dose regimen in a fasted state, considering C_τ_, pAUCs and HVD, are summarized in Table 2. Bioequivalence criteria are only met for the first of the two complementary partial AUCs.

### 3.2. Population Pharmacokinetic Model of Desvenlafaxine

A total number of 1275 desvenlafaxine measurements were included in the PK analysis from the cross-over randomized 2 × 2 bioequivalence study that was obtained from the 32 healthy volunteers that completed the study. Five samples (0.39%) were below the limit of quantification and were discarded from the analysis. A one-compartment PK model successfully described the observed desvenlafaxine data. The estimated total plasma apparent clearance (CL) for a typical healthy volunteer was 18.6 L/h and the apparent volume of distribution of the central compartment (V_1_) was estimated in 83.3 L. The absorption process after the oral administration of desvenlafaxine was described through a linear process (k_a_ = 7.37 × 10^−2^ h^−1^), assuming two transit compartments (k_tr_ = 18.5 h^−1^) in order to account for the delay in the appearance of desvenlafaxine measurable concentrations in plasma. Alternative mechanisms were also evaluated (i.e., zero-order absorption, non-linear kinetics, sequential/parallel first- and zero-order processes), but these resulted in a worse model performance (*p* > 0.05) (Supplementary Table S3).

Inter-individual random effects were associated with k_a_ (15%), k_tr_ (39%), CL (17%), and V_1_ (43%), showing a moderate IIV of PK parameters. IOV was incorporated on k_a_ (13%), k_tr_ (27%), and CL (14%). A non-diagonal element was explored, but did not statistically improve the model performance. The RUV was 18% using an additive error model for log-transformed observations.

Table 3 lists the estimates of the PK model parameters, together with their corresponding precision. All PK parameters were estimated with good precision, and 95% CI did not include null value.

**Figure 2:**
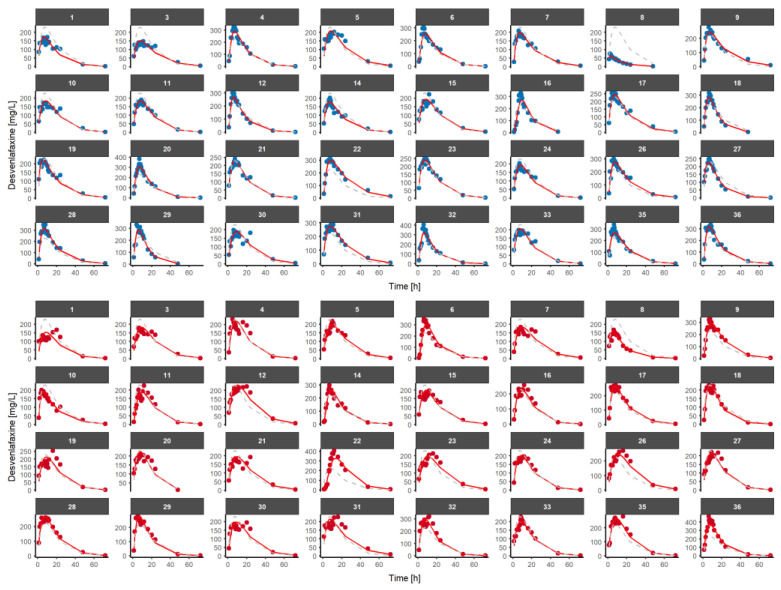
Individual predicted plots of the final population pharmacokinetic model of desvenlafaxine after the administration of a single dose of 100 mg of desvenlafaxine prolonged-release tablets to healthy volunteers in a fasted state. Blue dots represent desvenlafaxine observations from the Reference formulation and red dots represent desvenlafaxine observations from the Test formulation. Red line represents the individual predicted concentrations. Dashed grey line represents the population predicted concentrations.

**Figure 3:**
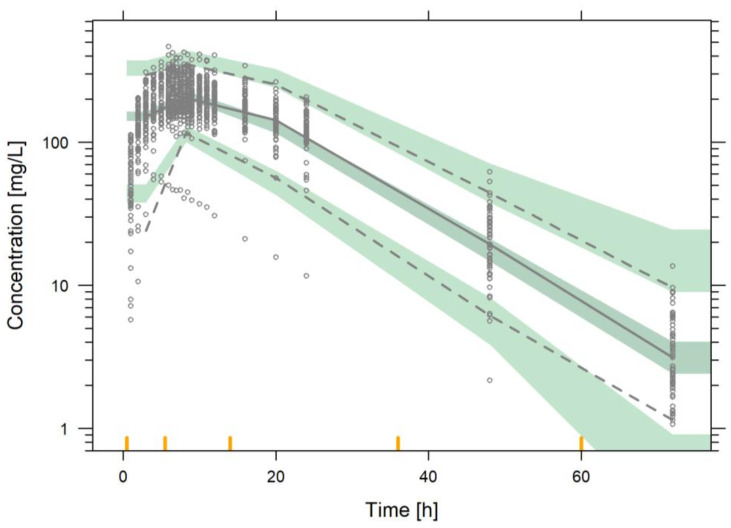
Visual Predictive check of the final population pharmacokinetic model for 100 mg of desvenlafaxine prolonged-release tablets after a single dose in healthy volunteers. Grey lines represent the 2.5th, 50th and 97.5th experimental percentiles. Grey shaded areas represent the 95% prediction interval of the 2.5th, 50th and 95th percentiles. Empty grey dots represent the experimental desvenlafaxine observations.

The results from the model evaluation exercise indicate that the model was capable of capturing the longitudinal profiles of the median, and the dispersion of the data based on the individual predicted PK profiles (Figure 2) and the pc-VPC (Figure 3).

### 3.3. Multiple-Dose Fasted-State Study Using a Model-Based Approach

**Figure 4:**
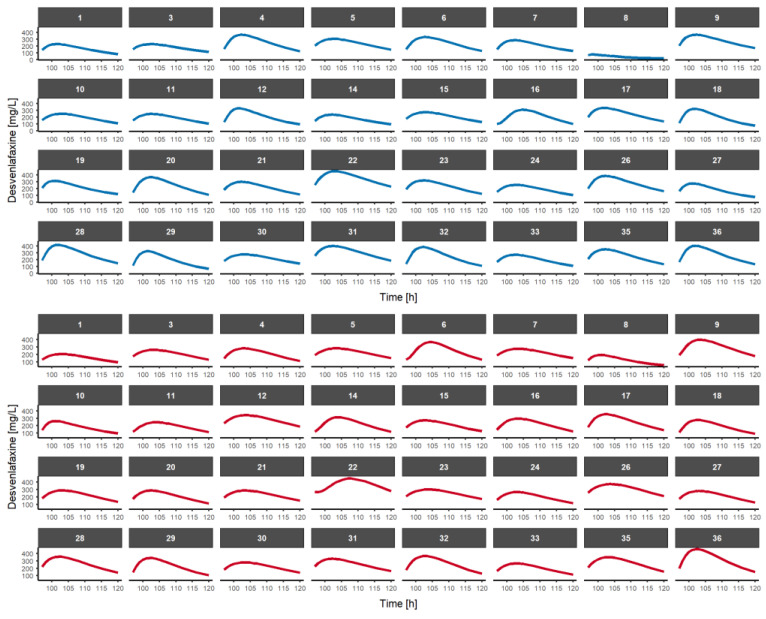
Individual pharmacokinetic time-course profiles at steady-state conditions of desvenlafaxine in healthy volunteers receiving 100 mg every 24 h. Blue and red lines represent desvenlafaxine simulated concentrations from Reference and Test formulations, respectively.

Figure 4 depicts a model-based simulation of desvenlafaxine after a multiple-dose regimen every 24 h at steady-state conditions. Individual estimated PK parameters from the single-dose study were used to simulate the PK profiles at steady-state conditions. The exposure metrics at steady state were derived using individual predicted concentrations and they do not consider the influence of residual error from the single-dose regimen. 

Individual exposure metrics (AUC_0-τ_, C_max,ss_ and C_τ,ss_) were numerically derived from the simulation analysis (Supplementary Table S4). Table 4 summarizes the bioequivalence assessment of desvenlafaxine for the simulated multiple-dose fasted-state study, showing that the bioequivalence criteria are not met because of C_τ,ss_.

## 4. Discussion

This single-dose bioequivalence study in the fasted state shows that generic prolonged-release products may produce a differently shaped plasma concentration–time curve relative to that of the reference medicinal product (Figure 1); it also shows that this different shape is not detected by the conventional PK metrics, i.e., C_max_, AUC_0-t_ and AUC_0-inf_ (Table 1), which are adequate only for single-dose bioequivalence studies of immediate release products. Due to this different shape in the plasma concentration–time curves, the simulated trough concentrations at steady state are not equivalent, which may be clinically relevant. To ensure that the test and reference prolonged-release products exhibit overlapping concentration–time curves, it is essential to confirm the bioequivalence of prolonged-release products at steady state when these prolonged-release products exhibit significant accumulation.

In the present case, the sponsor did not perform the bioequivalence study at steady state because the failure was anticipated, based on the single-dose results, and the development of this formulation was stopped. Unfortunately, therefore, the experimental evidence at steady state is not available, but it is understandable. However, our simulation confirmed the prediction conducted by the sponsor. C_τ,ss_ was significantly different between test and reference, as the 90% CI does not include the 100% value (GMR: 123.41%) and failed to show bioequivalence with a 90% CI of 111.08–137.10%. This notable difference did not prevent a conclusion of bioequivalence in the single-dose study, as shown in Table 1, although an approximate 10% difference is detected by the AUCs in the single-dose study; this shows that AUC_0-t_ or AUC_0-inf_ are not sensitive enough. The same problem has been detected in one-third of the evaluated prolonged-release products submitted to ANVISA/Brazil [28].

As it is ethically and financially desirable to avoid the conductance of multiple-dose bioequivalence studies, the identification of additional metrics in the single-dose study that are able to predict the outcome of the bioequivalence study at steady state is of great interest. In this regard, C_τ_, HVD and all the investigated pairs of pAUCs were able to detect the different shapes of the curves after a single dose and the bioequivalence failure of the predicted C_τ,ss_ in the simulated steady-state study.

It is not surprising that C_τ_ was able to predict the outcome of C_τ,ss_ because the simulation assumed linear pharmacokinetics, as described previously [17]. Interestingly, C_τ_ was not only slightly more sensitive to detecting the differences based on the point estimate than C_τ,ss_, as intuitively expected, since single-dose studies are generally considered more discriminative than multiple-dose studies, but it was also more sensitive than all the investigated pAUCs. Furthermore, its variability was not high (26.06%), although it was higher than those of the C_max_ (19.21%), AUC_0-t_ (15.97%) and AUC_0-inf_ (16.01%). Only the terminal pAUCs with only three and four samples for their calculation were slightly more variable than C_τ_ (27.97% and 26.44%, respectively); this may be due to five profiles where the sample at 72 h had levels below the lower limit of quantification, where the previous sample at 48 h had to be considered as the last sample with measurable concentrations for the terminal pAUC calculation. Therefore, pAUCs with a great imbalance in the number of samples used to calculate the two pAUCs do not offer any advantage over C_τ_ in this case, since they are slightly more variable than C_τ_ and less sensitive to detecting the existing difference. The terminal pAUC calculated with five samples (i.e., pAUC_16h-t_) was as sensitive to detecting the difference and was as variable as the simulated C_τ,ss_. In the present case, for cut-off points lower than 16 h, the larger the number of samples used to calculate the terminal pAUC was, the less sensitive and less variable these terminal pAUCs were.

The HVD GMR was as sensitive as that of pAUC_16-t_ and the simulated C_τ,ss_, and almost as sensitive as that of C_τ_, but HVD was slightly more variable than these parameters and more complex to calculate, since it implies interpolation. Therefore, in this case, HVD does not seem to provide any advantage.

These results suggest that C_τ_, carefully selected pAUCs and HVD might predict the bioequivalence outcome of the multiple-dose study required for prolonged-release products with accumulation. The differences detected by their GMR are similar and all of them are adequately sensitive to predict that the products are not bioequivalent at steady state. However, many more case studies are necessary to confirm whether these additional single-dose PK metrics are predictive of AUC_τ,ss_, C_max,ss_ and C_τ,ss_; this would require not only successful bioequivalence studies, but also real failed studies, which are much more difficult to obtain. This is especially the case in countries were multiple-dose studies are not required for regulatory approval. At least in these countries, the multiple-dose study could be simulated, as has been performed in the present work, to predict whether the bioequivalence at steady state can be expected under the study assumptions. If the predictions show that bioequivalence is not expected, the regulatory requirements should be reconsidered. Modelling and simulations would be useful to identify whether the regulations are failing to protect Public Health [29]; these would take into account that the problem may be worse, since the reality may be more complex than what can be expected from pharmacokinetic linearity, as shown previously [18].

Multiple-dose studies have been required in Canada in the past for these prolonged-release products with accumulation, but this requirement was removed from the regulations [19]. The lack of failed multiple-dose studies cannot justify this change in regulation, because studies are not submitted for regulatory approval if failed. More importantly, the absence of evidence is not evidence of absence.

## 5. Conclusions

In conclusion, this work adds another piece of evidence to those already available [17,18] to support that the conventional PK metrics for single-dose studies (C_max_; AUC_0-t_ and AUC_0-inf_) may not be enough to guarantee bioequivalence at steady state for prolonged-release products.

## Figures and Tables

**Table 1:** Bioequivalence evaluation of desvenlafaxine 100 mg prolonged-release tablets after a single dose under fasting conditions in 32 healthy volunteers.

PK Parameter	Point Estimate (%)	Lower Limit of 90% CI (%)	Upper Limit of 90% CI (%)	Intra-Subject CV (%)	BE Conclusion
AUC_0-t_	111.67	104.39	119.45	15.97	Yes
AUC_0-inf_ ^1^	111.33	103.97	119.22	15.95	Yes
C_max_	97.29	89.74	105.48	19.21	Yes

^1^ For AUC_0-inf_ only 31 subjects could be considered because extrapolation was not possible for subject #21. BE: bioequivalence; CI: confidence interval; CV: coefficient of variation; PK: pharmacokinetic.

**Table 2:** Bioequivalence evaluation of C_τ_ and different pAUCs of desvenlafaxine 100 mg prolonged-release tablets after the administration of a single dose under fasting conditions in 32 healthy volunteers.

PK Parameter	Point Estimate (%)	Lower Limit of 90% CI (%)	Upper Limit of 90% CI (%)	Intra-Subject CV (%)	BE Conclusion
C_τ_	125.51	112.57	139.93	26.06	No
pAUC_0–8h_	90.18	83.36	97.56	18.69	Yes
pAUC_8h-t_	118.68	109.37	128.70	19.35	No
pAUC_0–10h_	92.05	85.39	99.24	17.86	Yes
pAUC_10h-t_	121.23	111.21	132.14	20.53	No
pAUC_0–12h_	94.91	88.14	102.19	17.56	Yes
pAUC_12h-t_	122.91	112.17	134.67	21.79	No
pAUC_0–16h_	101.08	94.30	108.33	16.45	Yes
pAUC_16h-t_	123.70	111.78	136.89	24.22	No
pAUC_0–20h_	106.04	99.29	113.24	15.58	Yes
pAUC_20-t_	122.11	109.36	136.35	26.44	No
pAUC_0–24h_	108.90	102.15	116.09	15.16	Yes
pAUC_24h-t_	120.14	106.93	134.97	27.97	No
HVD	123.79	110.19	139.07	27.96	No

BE: bioequivalence; CI: confidence interval; CV: coefficient of variation; PK: pharmacokinetic.

**Table 3:** Final population pharmacokinetic parameters of 100 mg of desvenlafaxine in healthy volunteers after single-dose regimen.

	Population PK Model Estimates			Bootstrap Results		
*Fixed effect*	Value	RSE (%)	Shrinkage (%)	Median	RSE (%)	95%CI
k_a_ (h^–1^)	7.37 × 10^–2^	21		7.35 × 10^–2^	22	(6.84–7.56) × 10^–2^
k_tr_ (h^–1^)	18.50	31		18.78	32	(16.75–24.32)
CL (L/h)	18.65	12		18.20	14	(11.42–26.98)
V_1_ (L)	83.31	19		82.92	17	(65.31–101.64)
***Inter-individual variability***						
k_a_ (%)	15	19	21	15	20	(14–28)
k_tr_ (%)	39	31	29	38	33	(22–51)
CL (%)	17	12	10	17	13	(10–31)
V_1_ (%)	43	22	16	42	21	(30–58)
***Inter-occasion variability***						
k_a_ (%)	13	9	16	14	11	(7–33)
k_tr_ (%)	27	8	26	26	10	(19–41)
CL (%)	14	10	12	14	9	(8–21)
***Residual unexplained variability***						
Plasma (%)	18	7	8	17	8	(11–23)

k_a_: first-order absorption rate constant; k_tr_: first-order transit rate constant; CL: clearance; V_1_: apparent central volume of the central compartment; RSE: relative standard error; CI: confidence interval.

**Table 4:** Bioequivalence evaluation of desvenlafaxine 100 mg prolonged-release tablets at the simulated steady state, after a once-daily schedule in fasted state in 32 healthy volunteers.

PK Parameter	Point Estimate (%)	Lower Limit of 90% CI (%)	Upper Limit of 90% CI (%)	Intra-Subject CV (%)	BE Conclusion
AUC_0-τ_	106.37	99.30	113.94	16.31	Yes
C_max,ss_	102.79	95.28	110.89	18.02	Yes
C_τ,ss_	123.41	111.08	137.10	25.18	No

BE: bioequivalence; CI: confidence interval; CV: coefficient of variation; PK: pharmacokinetic.

## Data Availability

The data presented in this study are available on request from the corresponding author. The data are not publicly available due to legal restrictions.

## Supplementary Materials

The following supporting information can be downloaded at: https://www.mdpi.com/article/10.3390/pharmaceutics15020409/s1, Table S1: Individual AUC_0-t_, AUC_0-inf_ and C_max_ values for each subject and treatment; Table S2a–e: Individual values of C_τ_, pAUC_0–8h_, pAUC_8h-t_, pAUC_0–10h_, pAUC_10h-t_, pAUC_0–12h_, pAUC_12h-t_, pAUC_0–16h_, pAUC_16h-t_, pAUC_0–20h_, pAUC_20h-t_, pAUC_0–24h_, pAUC_24h-t_ and HVD for each subject and treatment. Table S3: List of models tested during the model building process. Table S4: Individual exposure metrics derived from the simulation analysis for each subject and treatment.
