# Supplementary material for: Alternative Pharmacokinetic Metrics in Single-Dose Studies to Ensure Bioequivalence of Prolonged-Release Products at Steady State—A Case Study
Source: Pharmaceutics. 2023 Jan 26;15(2):409. doi: 10.3390/pharmaceutics15020409 (PMC9963605; doi:10.3390/pharmaceutics15020409)
Supplement: Supplementary file 1 [file pharmaceutics-15-00409-s001.zip › pharmaceutics-2132640-supplementary.pdf]

TTITLE PAGE

Title: Alternative Pharmacokinetic Metrics in Single-Dose Studies to Ensure Bioequivalence of Prolonged-Release Products at Steady State—A Case Study

Authors: Víctor Mangas-Sanjuán [1,2,*], Marta Simón [3], Esperanza González-Rojano [4], Dolores Ochoa [5,6], Francisco Abad-Santos [5,6], Manuel Román [5], Mercedes Ramos [3], Carlos Govantes [3] and Alfredo García-Arieta [7]

Affiliation:

[1] Department of Pharmacy and Pharmaceutical Technology and Parasitology, University of Valencia, 46100 Valencia, Spain

[2] Interuniversity Research Institute for Molecular Recognition and Technological Development, Polytechnic University of Valencia—University of Valencia, 46100 Valencia, Spain

[3] Laboratorios Normon, 28760 Madrid, Spain

[4] Clinical Pharmacology Department, Hospital Universitario Clínico San Carlos, Instituto de Investigación Sa-nitaria del Hospital Clínico San Carlos (IdISSC), 28040 Madrid, Spain

[5] Clinical Pharmacology Department, Hospital Universitario de La Princesa, Instituto Teófilo Hernando, Insti-tuto de Investigación Sanitaria la Princesa (IIS-IP), 28006 Madrid, Spain

[6] Pharmacology Department, Facultad de Medicina, Universidad Autónoma de Madrid, 28029 Madrid, Spain

[7] División de Farmacología y Evaluación Clínica, Departamento de Medicamentos de Uso Humano, Agencia Española de Medicamentos y Productos Sanitarios, 28022 Madrid, Spain

Corresponding author: Víctor Mangas-Sanjuán

E-mail: victor.mangas@uv.es

Telephone: +34-963-543-351

Supplementary Table S1. Individual AUC0-t, AUC0-inf and Cmax values for each subject and treatment.

Subject	Sequence	Test C_{max}	Ref. C_{max}	Test AUC_{0-t}	Ref. AUC_{0-t}	Test AUC_{0-inf}	Ref. AUC_{0-inf}
1	RT	167.08	172.24	4947.005	4571.595	4962.20	4596.10
3	RT	178.66	147.89	5759.4925	4989.6025	5809.73	5070.26
4	TR	231.17	325.08	6282.96	6380.9475	6295.83	6412.05
5	TR	218.28	202.48	6081.9025	6677.4125	6144.24	6768.88
6	TR	349.84	305.22	6215.03	6354.105	6239.84	6387.77
7	TR	183.12	207.86	6109.5725	5490.8125	6207.74	5590.06
8	RT	167.31	74.63	3079.7725	962.8875	3096.41	993.31
9	TR	325.67	278.37	7602.97	6873.57	7692.76	7077.25
10	RT	199.74	172.63	4980.9275	5542.5925	5033.90	5570.00
11	RT	225.81	190.26	5304.1425	4941.07	5325.24	4974.42
12	TR	222.31	299.75	7722.0375	5246.1925	7809.74	5276.49
14	RT	299.93	200.32	5820.835	4687.1825	5832.56	4717.90
15	RT	196.25	222.24	5955.345	5706.9925	6000.00	5764.90
16	RT	257.39	330.18	6383.9525	5165.5175	6396.39	5258.31
17	TR	274.42	254.68	6603.7725	6841.17	6672.72	6912.87
18	TR	229.80	318.00	5108.5225	4876.0275	5120.10	4943.25
19	RT	252.98	223.10	6854.115	6119.785	6881.77	6181.10
20	TR	213.52	384.11	5804.6325	6325.7875	5916.59	6340.03
21	RT	193.11	242.03	6751.4325	5881.9275		5908.76
22	TR	404.91	311.66	9230.57	8731.8325	9364.43	9023.98
23	RT	212.79	248.41	6938.2725	6104.0325	7034.21	6147.22
24	TR	196.32	203.11	5573.3725	5242.8125	5598.51	5264.99
26	RT	269.13	300.16	8416.6175	7436.935	8539.78	7562.14
27	TR	222.06	249.25	5931.785	4342.500	5954.54	4356.58
28	RT	260.08	351.37	6966.5825	7542.4725	7016.25	7606.68
29	RT	276.43	331.84	6331.3925	4833.3525	6350.20	4891.67
30	TR	194.44	196.95	6321.715	6336.545	6372.82	6414.36
31	TR	226.61	287.04	7531.8275	7849.1725	7676.05	8014.07
32	TR	318.93	407.24	7263.2625	6425.135	7277.28	6444.71
33	RT	227.10	197.58	5268.09	5801.425	5294.77	5833.53
35	RT	280.03	338.38	7398.3175	6514.9575	7422.43	6558.65
36	TR	464.76	336.47	7755.5775	7081.4375	7786.43	7122.55

Supplementary Table S2a. Individual values of Cτ, pAUC0-8h and pAUC0-8h-t for each subject and treatment.

Subject	Test C_τ	Ref. C_τ	Test $AUC_{0\text{-}8h}$	Ref. $AUC_{0\text{-}8h}$	Test $AUC_{8h\text{-}t}$	Ref. $AUC_{8h\text{-}t}$
1	126.05	103.03	851.11	1043.07	4095.89	3528.53
3	138.27	121.28	988.13	859.22	4771.37	4130.38
4	149.66	105.36	1290.53	1695.74	4992.44	4685.21
5	135.57	148.8	990.24	1134.58	5091.67	5542.84
6	110.84	131.46	1465.49	1491.98	4749.54	4862.13
7	158.55	106.42	1017.70	1153.57	5091.87	4337.24
8	45.9	11.62	978.25	416.23	2101.52	546.66
9	150.38	110.15	1495.09	1471.19	6107.89	5402.38
10	102.1	137.86	1199.60	1037.39	3781.33	4505.21
11	116.78	100.07	877.98	1044.98	4426.17	3896.09
12	187.15	71.82	1195.25	1621.76	6526.79	3624.43
14	121.45	98.17	1227.60	1088.51	4593.23	3598.68
15	129.68	114.08	1120.15	1052.79	4835.20	4654.20
16	135.32	97.35	1191.89	1092.10	5192.06	4073.42
17	112.12	140.45	1537.43	1528.26	5066.34	5312.91
18	90.28	59.21	1267.22	1625.26	3841.30	3250.77
19	164.96	135.07	1163.49	1456.99	5690.63	4662.80
20	128.3	112.75	1250.04	1720.89	4554.59	4604.90
21	193.11	128.81	1052.01	1348.87	5699.42	4533.06
22	207.26	147.69	720.51	1664.87	8510.07	7066.97
23	161.52	118.7	1001.21	1453.82	5937.07	4650.21
24	117.45	119.3	1116.00	1120.58	4457.38	4122.24
26	198.01	155.4	1233.36	1653.62	7183.26	5783.32
27	116.15	54.49	1089.20	1417.02	4842.58	2925.48
28	128.3	139.19	1611.72	1911.18	5354.87	5631.29
29	114.64	56.74	1671.35	1723.87	4660.04	3109.48
30	157.59	181.45	1125.61	1039.11	5196.11	5297.43
31	166.81	138.85	1252.68	1648.48	6279.15	6200.69
32	124.64	109.97	1696.63	1858.98	5566.63	4566.16
33	102.52	131.66	1046.98	1204.78	4221.11	4596.64
35	151.5	108.6	1457.02	1750.52	5941.30	4764.44
36	108.66	126.08	2105.00	1865.13	5650.58	5216.31

Supplementary Table S2b. Individual values of pAUC0-10h and pAUC10h-t for each subject and treatment.

Subject	Test AUC_{0-10h}	Ref. AUC_{0-10h}	Test AUC_{10-t}	Ref. AUC_{10-t}	Test AUC_{0-12h}	Ref. AUC_{0-12h}
1	1078.74	1337.16	3868.27	3234.44	1323.05	1633.38
3	1311.53	1125.41	4447.97	3864.20	1620.97	1400.00
4	1648.66	2262.72	4634.30	4118.23	1972.06	2706.09
5	1387.51	1503.74	4694.39	5173.68	1800.80	1894.61
6	2091.86	1960.44	4123.17	4393.67	2637.53	2384.07
7	1358.11	1517.72	4751.46	3973.10	1663.99	1859.83
8	1256.16	496.78	1823.62	466.11	1515.33	565.85
9	2069.66	1949.63	5533.32	4923.95	2577.15	2377.33
10	1534.41	1366.70	3446.52	4175.89	1828.55	1662.97
11	1173.64	1405.97	4130.50	3535.11	1554.20	1736.43
12	1591.12	2099.28	6130.92	3146.91	1998.24	2533.73
14	1735.79	1419.29	4085.05	3267.90	2185.66	1692.96
15	1469.30	1385.89	4486.05	4321.10	1825.85	1751.35
16	1590.65	1713.26	4793.30	3452.26	2058.37	2198.62
17	2053.95	1974.49	4549.83	4866.69	2536.25	2368.09
18	1666.97	2158.65	3441.56	2717.38	2084.10	2619.11
19	1509.16	1852.20	5344.96	4267.59	1834.14	2185.71
20	1664.81	2321.20	4139.83	4004.59	2056.91	2820.63
21	1401.16	1780.95	5350.27	4100.98	1733.21	2207.01
22	1397.50	2250.38	7833.08	6481.45	2163.91	2802.43
23	1317.36	1919.08	5620.92	4184.95	1705.53	2354.61
24	1478.70	1459.00	4094.68	3783.82	1865.51	1776.25
26	1661.36	2162.85	6755.26	5274.09	2158.22	2608.58
27	1508.08	1852.28	4423.71	2490.22	1906.91	2229.20
28	2106.45	2494.64	4860.13	5047.83	2598.94	3026.01
29	2124.61	2255.49	4206.78	2577.87	2599.29	2722.59
30	1460.29	1396.49	4861.43	4940.06	1770.92	1741.67
31	1592.70	2174.08	5939.13	5675.09	1986.07	2722.79
32	2188.98	2488.78	5074.28	3936.36	2790.52	2976.72
33	1481.31	1563.54	3786.79	4237.89	1876.45	1926.01
35	1956.45	2262.10	5441.87	4252.86	2430.18	2700.04
36	2903.55	2494.42	4852.03	4587.02	3629.50	3006.36

Supplementary Table S2c. Individual values of pAUC0-12h, pAUC12h-t, pAUC0-16h and pAUC16h-t for each subject and treatment.

Subject	Test AUC_{12h-t}	Ref. AUC_{12h-t}	Test AUC_{0-16h}	Ref. AUC_{0-16h}	Test AUC_{16h-t}	Ref. AUC_{16h-t}
1	3623.96	2938.22	1860.87	2129.52	3086.14	2442.08
3	4138.52	3589.60	2195.45	1887.62	3564.04	3101.98
4	4310.90	3674.86	2694.98	3468.01	3587.98	2912.94
5	4281.10	4782.80	2513.18	2671.43	3568.72	4005.98
6	3577.50	3970.04	3449.01	3120.07	2766.02	3234.04
7	4445.58	3630.98	2216.35	2441.67	3893.22	3049.14
8	1564.44	397.04	1900.17	669.13	1179.6	293.76
9	5025.82	4496.24	3515.53	3095.59	4087.44	3777.98
10	3152.38	3879.62	2332.09	2241.37	2648.84	3301.22
11	3749.94	3204.64	2373.36	2344.95	2930.78	2596.12
12	5723.80	2712.46	2866.94	3233.47	4855.1	2012.72
14	3635.18	2994.22	2866.24	2160.20	2954.6	2526.98
15	4129.50	3955.64	2562.87	2556.41	3392.48	3150.58
16	4325.58	2966.90	3024.07	2951.36	3359.88	2214.16
17	4067.52	4473.08	3421.21	3074.25	3182.56	3766.92
18	3024.42	2256.92	2811.72	3317.81	2296.8	1558.22
19	5019.98	3934.08	2731.84	2759.87	4122.28	3359.92
20	3747.72	3505.16	2788.29	3586.01	3016.34	2739.78
21	5018.22	3674.92	2310.11	2892.49	4441.32	2989.44
22	7066.66	5929.40	3659.69	3766.25	5570.88	4965.58
23	5232.74	3749.42	2540.73	3052.01	4397.54	3052.02
24	3707.86	3466.56	2622.99	2394.67	2950.38	2848.14
26	6258.40	4828.36	3221.14	3443.78	5195.48	3993.16
27	4024.88	2113.30	2745.61	2812.04	3186.18	1530.46
28	4367.64	4516.46	3476.16	3911.33	3490.42	3631.14
29	3732.10	2110.76	3457.99	3388.25	2873.4	1445.1
30	4550.80	4594.88	2423.40	2292.67	3898.32	4043.88
31	5545.76	5126.38	2885.15	3636.79	4646.68	4212.38
32	4472.74	3448.42	3951.62	3731.22	3311.64	2693.92
33	3391.64	3875.42	2575.85	2637.25	2692.24	3164.18
35	4968.14	3814.92	3455.66	3476.46	3942.66	3038.5
36	4126.08	4075.08	4697.72	3744.30	3057.86	3337.14

Supplementary Table S2d. Individual values of pAUC0-20h, pAUC20h-t and pAUC0-24h for each subject and treatment.

Subject	Test AUC_{0-20h}	Ref. AUC_{0-20h}	Test AUC_{20h-t}	Ref. AUC_{20h-t}	Test AUC_{0-24h}	Ref. AUC_{0-24h}
1	2500.63	2563.14	2446.38	2008.46	3086.89	2994.56
3	2795.37	2357.46	2964.12	2632.14	3386.13	2823.24
4	3515.22	4163.19	2767.74	2217.76	4205.16	4692.07
5	3135.24	3407.45	2946.66	3269.96	3702.90	4069.21
6	4049.01	3753.43	2166.02	2600.68	4512.71	4308.59
7	2821.43	2959.01	3288.14	2531.8	3477.01	3438.81
8	2161.19	742.79	918.58	220.1	2365.77	797.53
9	4348.39	3680.27	3254.58	3193.3	5008.09	4161.09
10	2724.65	2763.05	2256.28	2779.54	3088.65	3275.91
11	3052.24	2867.39	2251.9	2073.68	3597.14	3317.23
12	3747.96	3724.73	3974.08	1521.46	4566.88	4075.71
14	3465.88	2584.44	2354.96	2102.74	4022.16	2965.06
15	3188.87	3185.53	2766.48	2521.46	3729.71	3682.23
16	3816.51	3491.56	2567.44	1673.96	4428.67	3896.40
17	4114.03	3657.21	2489.74	3183.96	4664.69	4189.65
18	3368.30	3791.67	1740.22	1084.36	3780.48	4089.55
19	3645.06	3266.01	3209.06	2853.78	4382.24	3774.39
20	3525.77	4186.69	2278.86	2139.1	4131.35	4688.87
21	2878.55	3417.19	3872.88	2464.74	3580.55	3918.85
22	4874.21	4614.51	4356.36	4117.32	5817.29	5303.67
23	3352.93	3662.57	3585.34	2441.46	4062.59	4185.47
24	3283.85	2955.09	2289.52	2287.72	3812.49	3443.65
26	4230.10	4153.56	4186.52	3283.38	5096.82	4781.34
27	3545.75	3212.74	2386.04	1129.76	4142.95	3478.86
28	4185.12	4578.39	2781.46	2964.08	4756.78	5140.55
29	4128.37	3800.97	2203.02	1032.38	4651.99	4085.03
30	3158.62	2796.57	3163.1	3539.98	3862.68	3429.19
31	3705.17	4395.29	3826.66	3453.88	4405.59	5011.65
32	4843.04	4319.08	2420.22	2106.06	5460.50	4794.94
33	3173.83	3236.83	2094.26	2564.6	3653.61	3748.23
35	4401.24	4117.14	2997.08	2397.82	5089.76	4595.68
36	5496.86	4401.12	2258.72	2680.32	6053.02	4980.00

Supplementary Table S2e. Individual values of pAUC24h-t and HVD for each subject and treatment.

Subject	Test AUC_{24h-t}	Ref. AUC_{24h-t}	Test HVD	Ref. HVD
1	1860.12	1577.04	32.25	27.49
3	2373.36	2166.36	33.25	34.86
4	2077.80	1688.88	28.18	17.04
5	2379.00	2608.20	27.99	32.74
6	1702.32	2045.52	12.67	15.92
7	2632.56	2052.00	34.17	23.05
8	714.00	165.36	13.93	9.05
9	2594.88	2712.48	19.19	16.67
10	1892.28	2266.68	17.38	33.63
11	1707.00	1623.84	20.85	23.74
12	3155.16	1170.48	34.28	13.22
14	1798.68	1722.12	14.38	17.18
15	2225.64	2024.76	29.93	23.11
16	1955.28	1269.12	22.90	11.02
17	1939.08	2651.52	19.87	24.91
18	1328.04	786.48	17.89	12.70
19	2471.88	2345.40	28.75	28.26
20	1673.28	1636.92	27.24	11.19
21	3170.88	1963.08	36.85	24.12
22	3413.28	3428.16	17.81	20.78
23	2875.68	1918.56	32.74	21.57
24	1760.88	1799.16	26.97	26.29
26	3319.80	2655.60	30.93	23.33
27	1788.84	863.64	23.35	14.13
28	2209.80	2401.92	22.39	15.41
29	1679.40	748.32	19.35	11.01
30	2459.04	2907.36	33.04	35.37
31	3126.24	2837.52	33.32	21.75
32	1802.76	1630.20	19.92	8.87
33	1614.48	2053.20	20.78	29.69
35	2308.56	1919.28	23.67	14.91
36	1702.56	2101.44	12.71	13.65

Supplementary Table S3. List of models tested during the model building process.

Model number	Description	Number of parameters	OFV	AIC	Comments	Statistical improvement (p<0.05)	Reference model
1	One-compartment model with linear kinetics	7	-1731	-1717	Minimization successful	-	-
2	Two-compartment model with linear kinetics	10	-1731	-1711	Minimization successful with rounding errors	No	1
3	One-compartment model with linear kinetics with lag time	9	-2131	-2113	Minimization successful	Yes	3
4	One compartment model with 1 transit compartment	9	-2178	-2160	Minimization successful	Yes	4
5	One compartment model with 2 transit compartments	9	-2392	-2374	Minimization successful	Yes	5
6	One compartment model with 3 transit compartments	9	-2394	-2376	Minimization successful	No	5
7	One compartment model with parallel absorption: zero-order and first-order with 2 transit compartments	13	-2394	-2376	Minimization terminated	No	5
**8**	**One compartment model with 2 transit compartments and inter-occasion variability for period effect**	**12**	**-2467**	**-2443**	**Minimization successful**	**Yes**	**8**
9	One compartment model with 2 transit compartments and formulation effect on ka	10	-2393	-2363	Minimization terminated	No	8
10	One compartment model with 2 transit compartments and formulation effect on ktr	10	-2393	-2363	Minimization terminated	No	8
11	One compartment model with 2 transit compartments	18	-2469	-2433	Minimization successful with rounding errors	No	8

	and inter-occasion variability for period effect and full omega block						

Supplementary Table S4. Individual exposure metrics derived from the simulation analysis for each subject and treatment.

Subject	Test $C_{max.ss}$	Ref. $C_{max.ss}$	Test $AUC_{0-\tau}$	Ref. $AUC_{0-\tau}$	Test $C_{\tau,ss}$	Ref. $C_{\tau,ss}$
1	248.79	263.78	3558.60	3779.03	88.19	64.32
3	332.15	282.99	4522.24	4023.48	86.99	96.27
4	379.02	447.96	4677.23	5997.85	116.61	117.87
5	346.51	412.57	5114.73	4882.94	124.82	142.69
6	489.61	434.11	5629.78	5759.53	135.97	130.18
7	405.37	305.55	5054.24	4469.84	169.37	149.77
8	265.15	93.23	2857.11	936.74	39.91	16.41
9	548.78	460.86	6759.93	6166.96	200.56	141.83
10	334.35	294.01	4195.44	3939.39	104.46	93.63
11	346.23	473.95	3985.18	4122.07	159.89	120.63
12	432.98	471.92	6476.18	5003.39	197.83	102.74
14	383.68	310.38	5108.16	3976.67	74.04	75.05
15	381.38	441.73	4853.23	5029.89	113.82	192.12
16	407.56	366.43	5510.30	4793.71	147.88	84.92
17	542.94	461.97	5818.34	5441.25	153.58	167.02
18	336.50	413.39	4457.96	5011.35	113.19	55.33
19	352.83	379.93	4635.60	5073.73	161.11	126.74
20	388.85	445.46	4956.58	5571.54	106.31	100.41
21	448.54	386.68	5175.85	4714.89	176.11	87.12
22	551.64	579.23	8170.74	8084.22	358.42	203.38
23	394.73	349.46	5092.87	5272.94	181.69	116.40
24	361.91	367.38	4569.22	4602.37	152.37	92.16
26	440.67	538.78	7016.53	6906.83	241.02	200.78
27	345.33	346.54	5164.26	3998.35	128.33	72.25
28	426.41	518.69	6029.41	6818.10	112.00	123.48
29	428.68	413.68	5330.13	4670.13	70.40	54.01
30	406.59	442.92	4702.14	5190.38	108.71	109.87
31	473.62	508.26	5618.97	6668.53	153.68	259.95
32	420.15	529.75	6099.43	5619.72	147.88	94.44
33	360.94	384.75	4571.78	4710.48	88.06	114.35
35	453.37	439.35	6054.93	5409.09	125.02	103.01
36	694.09	451.56	7785.66	6415.79	186.42	141.15
